# A deep learning approach for orphan gene identification in moso bamboo (*Phyllostachys edulis*) based on the CNN + Transformer model

**DOI:** 10.1186/s12859-022-04702-1

**Published:** 2022-05-05

**Authors:** Xiaodan Zhang, Jinxiang Xuan, Chensong Yao, Qijuan Gao, Lianglong Wang, Xiu Jin, Shaowen Li

**Affiliations:** 1 Anhui Province Key Laboratory of Smart Agricultural Technology and Equipment, Anhui Agriculture University, Hefei, 230001 China; 2 College of Information and Computer Science, Anhui Agricultural University, Hefei, 230001 China; 3 Graduate School, Anhui Agricultural University, Hefei, 230036 China

**Keywords:** Orphan genes, Moso bamboo, Deep learning, Convolutional neural network, Transformer neural network

## Abstract

**Background:**

Orphan gene play an important role in the environmental stresses of many species and their identification is a critical step to understand biological functions. Moso bamboo has high ecological, economic and cultural value. Studies have shown that the growth of moso bamboo is influenced by various stresses. Several traditional methods are time-consuming and inefficient. Hence, the development of efficient and high-accuracy computational methods for predicting orphan genes is of great significance.

**Results:**

In this paper, we propose a novel deep learning model (CNN + Transformer) for identifying orphan genes in moso bamboo. It uses a convolutional neural network in combination with a transformer neural network to capture k-mer amino acids and features between k-mer amino acids in protein sequences. The experimental results show that the average balance accuracy value of CNN + Transformer on moso bamboo dataset can reach 0.875, and the average Matthews Correlation Coefficient (MCC) value can reach 0.471. For the same testing set, the Balance Accuracy (BA), Geometric Mean (GM), Bookmaker Informedness (BM), and MCC values of the recurrent neural network, long short-term memory, gated recurrent unit, and transformer models are all lower than those of CNN + Transformer, which indicated that the model has the extensive ability for OG identification in moso bamboo.

**Conclusions:**

CNN + Transformer model is feasible and obtains the credible predictive results. It may also provide valuable references for other related research. As our knowledge, this is the first model to adopt the deep learning techniques for identifying orphan genes in plants.

**Supplementary Information:**

The online version contains supplementary material available at 10.1186/s12859-022-04702-1.

## Background

OG are genes that lack homologs in other lineages [1]. Thus, genes are generally classified as orphans if they lack coding-sequence similarity outside of their own species [2]. OG appear to be present in all species and make up 10% to 30% of all genes in a genome [3]. Currently, a growing number of OG are being identified in plants, including taxa such as *Arabidopsis*, *Populus*, *Oryza sativa* and *sweet orange* [4–7]. Many annotated OG are often differentially expressed in response to stresses and are considered to be determinant of species characteristics [8–10]. Hence, the identification of OG may provide a better understand for OG adaptation.

Moso bamboo (*Phyllostachys edulis*) belongs to the subfamily Bambusoideae of the Poaceae family; it shows characteristics of fast growth and excellent material production and can therefore be used to produce cloths, artwork, paper and food [11]. Recent studies have revealed that stresses such as drought and high temperature can affect the growth of moso bamboo as well as the yield and quality of moso bamboo shoots [12, 13]. Although the identification of OG has been widely carried out in many plant species*,* a comprehensive understanding of OG is lacking in moso bamboo. Therefore, the discovery of OG is of great significance for subsequent research in this species.

Currently, orphan genes are generally obtained through BLAST sequence alignment, which compares the sequencing sequence (genome sequence, transcriptome sequence, etc.) of the studied species with other species, BLAST is a relatively reliable tool for identifying orphan genes [2]. BLAST, including BLASTP and tBLASTn, are often used as the alignment tools [14–16]. However, the use of these methods to identify OG requires considerable server and time resources and is greatly affected by the computational approach applied [17]. Orphan genes are widely distributed in plant species and generally exhibit significant differences in gene length, the number of exons, GC content, and expression level compared to protein-coding genes [3, 6, 10, 16, 18]. Therefore, orphan genes and non-orphan genes are distinguishable in terms of protein features. In traditional machine learning methods, features are often selected and extracted manually, which requires researchers to have prior domain knowledge and keen insight into the relationships between gene essentiality and types of biological data to obtain informative features to train the models. For example, Ying et al. [19] employed a machine learning-based approach to predict autism spectrum disorder (ASD) risk genes using human brain spatiotemporal gene expression signatures, gene-level constraint metrics, and other gene variation features. Recent study shows that using deep learning to extract features often achieves better results compared to its closest machine learning competitors, the majority of these deep learning algorithms rely on features extracted from raw sequences [20]. Hence, it would be significant to develop an efficient method that uses only protein sequences to train such models and produces credible predictive results.

As deep learning has gained popularity, it has been applied successfully in many bioinformatics fields, such as gene prediction [21] and medical image segmentation [22]. In particular, deep learning technology is used to automatically extract and learn abstract information from data to train a model; this approach shows superior performance and high adaptability and avoids complex feature engineering in natural language processing [23]. Moreover, protein sequences are very similar to natural language; the amino acids in proteins are similar to the words in natural language, and the same contextual relationship exists between amino acids in a protein sequence and as between words in a sentence. In this context, the prediction of OG can be considered a natural language processing problem.

Recently, transformer models have been widely used to address sequence problems. Zheng et al. [24] proposed the Segmentation Transformer (SETR) model, which regards semantic segmentation as a sequence-to-sequence prediction task. Zou et al. [25] proposed a transformer model for end-to-end target detection.

In this paper, OG are predicted by using hybrid deep learning based on convolutional neural networks and transformer neural networks. The raw protein sequences are first encoded as vectors or matrices by encoder or word2vec encoding, respectively. Then, protein features are extracted by CNN and transformer model. Finally, the extracted features are input into the fully connected neural network to generate the final recognition result. CNN + Transformer only uses protein sequences to train the models to predict moso bamboo OG. It uses two multicore convolution layers to capture high-frequency k-mer features in protein sequences. The extracted k-mer features are provided to the transformer layer, which captures the long-term interaction information between k-mer features through a multi-head self-attention mechanism.

## Methods

### Dataset

**Fig. 1:**
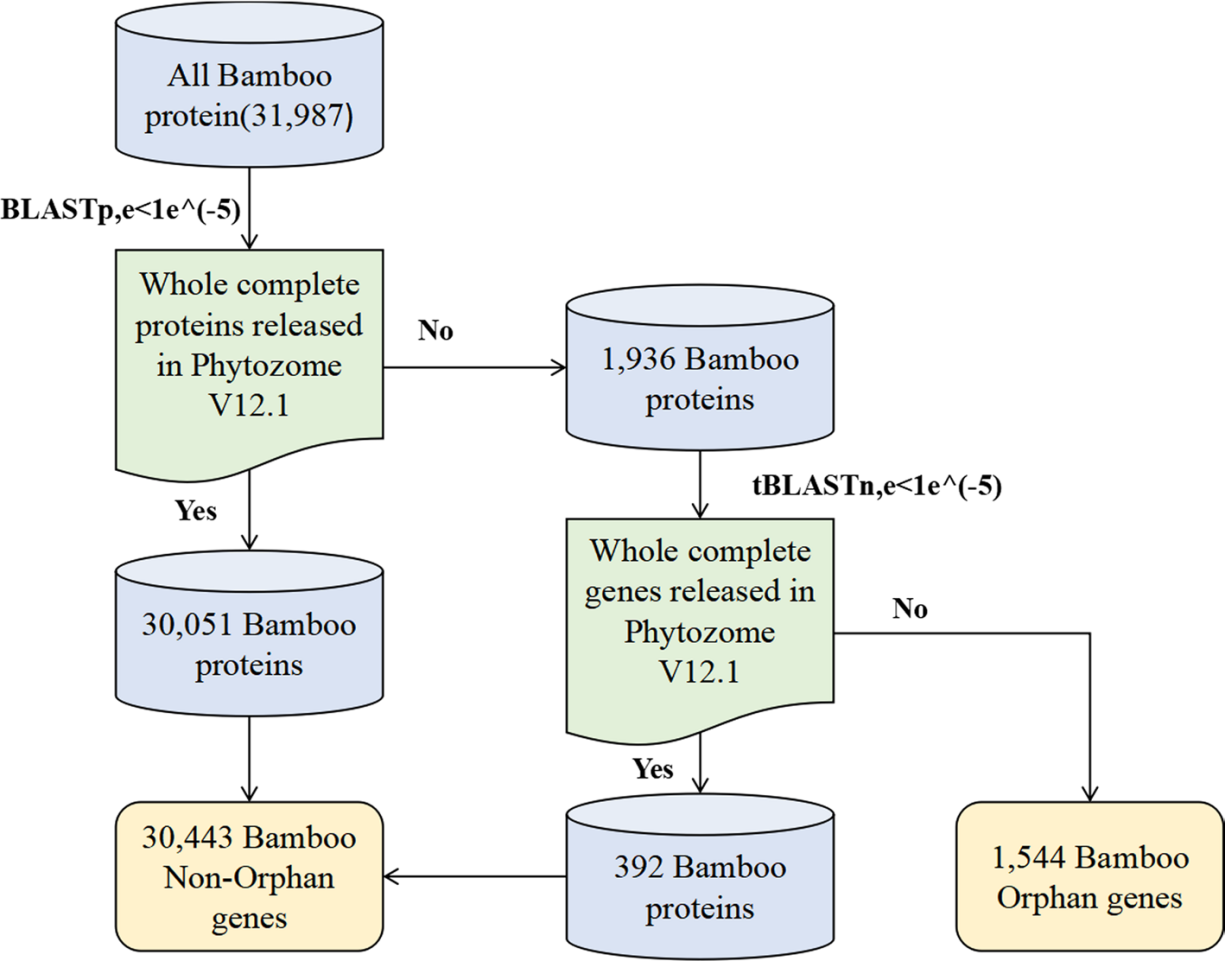
Flow chart of data acquisition for moso bamboo OG

BLAST (2.11.0+) [26] was used to identify OG based on previous studies [6, 27–29]. Moso bamboo protein sequences were downloaded from Bamboo GDB [30]. First, we used BLASTp to search for homologs of all 31,987 proteins annotated in moso bamboo in each of the other 136 plant species released in Phytozome v12.1 [31] with an e-value cutoff of 1*e*^−5^. A total of 30,443 moso bamboo genes showed significant similarity to at least one sequence, which were defined as Evolutionarily Conserved genes (ECs) [32] and removed from further analysis (Fig. 1). Second, the remaining 1936 moso bamboo proteins for which no homologs could be found in any of the genomes were used for the next step of searches, which was performed by tBLASTn analysis. In this step, 392 moso bamboo genes were classified as ECs. The final sets of ECs and OG contained 30,443 and 1,544 genes (Additional file 1: Table S1), respectively (Fig. 1).

To adequately train the deep learning model, the 1544 obtained OG were identified with label 1, and 30,443 ECs were identified with label 0. These genes were combined to form the moso bamboo orphan gene dataset.

### Protein embedding

Protein sequences consist of possible 20 amino acids, each of which is represented by a capital letter. To make the protein sequence recognizable by a computer, the first step is to encode each amino acid in the protein according to Table 1, mapping each amino acid to a specific real number, where values of 1–20 represent the amino acid types, and unspecified or unknown amino acids are denoted as 21 [33, 34]. The amino acid coding sequence in the table does not affect the experimental results. The sequence profiles thus obtained for each sequence search were processed by truncating the profiles of long sequences to a fixed length (*L*) and zero padding short sequences, a method that is widely used for data preprocessing and effective training [35]. As a result, we obtained a one-dimensional vector for each protein.

1 $$s = (s_{1} ,s_{2} , \ldots ,s_{L} )s_{i} \in \{ 0,1, \ldots ,21\}$$

**Table 1:** Amino acid embedding cross-reference table

Amino acids	Letters	Code	Amino acids	Letters	Code
Histidine	H	1	Methionine	M	2
Alanine	A	3	Lysine	K	4
Cysteine	C	5	Arginine	R	6
Lucine	L	7	Tyrosine	Y	8
Serine	S	9	Aspartic	D	10
Glycine	G	11	Valine	V	12
Isoleucine	I	13	Glutamic	E	14
Asparagine	N	15	Tryptophan	W	16
Phenylalanine	F	17	Threonine	T	18
Glutamine	Q	19	Proline	P	20
Illegal Amino acids	B,J,O,U,X,Z	21			

For protein sequences, by learning the dense continuous feature representation of each amino acid in the sequence, a distributional representation can be learned for the amino acids. When these embedding vectors are projected in 2D, it can be shown that amino acids with similarities in hydrophobicity, polarity and net charge, which are factors important for covalent chemical bonding, form visually distinguishable groups [36]. This validates the used of distributed representation as an effective method for encoding amino acids that also helps to preserve important physiochemical properties.

Hence, the sparse feature vectors (*S*) of a given protein sequence are transformed to dense continuous feature representations using word embedding transformation as follows:$F_{e}^{1} \in {\mathbb{R}}^{L \times e}$, where *e* corresponds to the embedding dimension. The self-attention mechanism in the transformer cannot distinguish between words at different positions, so the input sequence needs to be position-encoded to incorporate the positional information into the input sequence. The input sequence is then encoded with a two-dimensional matrix, $F_{e}^{2} \in {\mathbb{R}}^{L \times e}$. $F_{e}^{1}$ and $F_{e}^{2}$ are added together as the input of CNN + Transformer, as follows:

2 $$F_{e} = F_{e}^{1} + F_{e}^{2} = \left[ {\begin{array}{*{20}c} {s_{1,1} } & \cdots & {s_{1,j} } & \cdots & {s_{1,e} } \\ \vdots & \cdots & \vdots & \cdots & \vdots \\ {s_{i,1} } & \cdots & {s_{i,j} } & \cdots & {s_{i,e} } \\ \vdots & \cdots & \vdots & \cdots & \vdots \\ {s_{L,1} } & \cdots & {s_{L,j} } & \cdots & {s_{L,e} } \\ \end{array} } \right]$$

Here, $S_{i,j}$ corresponds to the $j^{th}$ word embedding number of the $i^{th}$ amino acid of the protein sequence after preprocessing.

### Transformer model

**Fig. 2:**
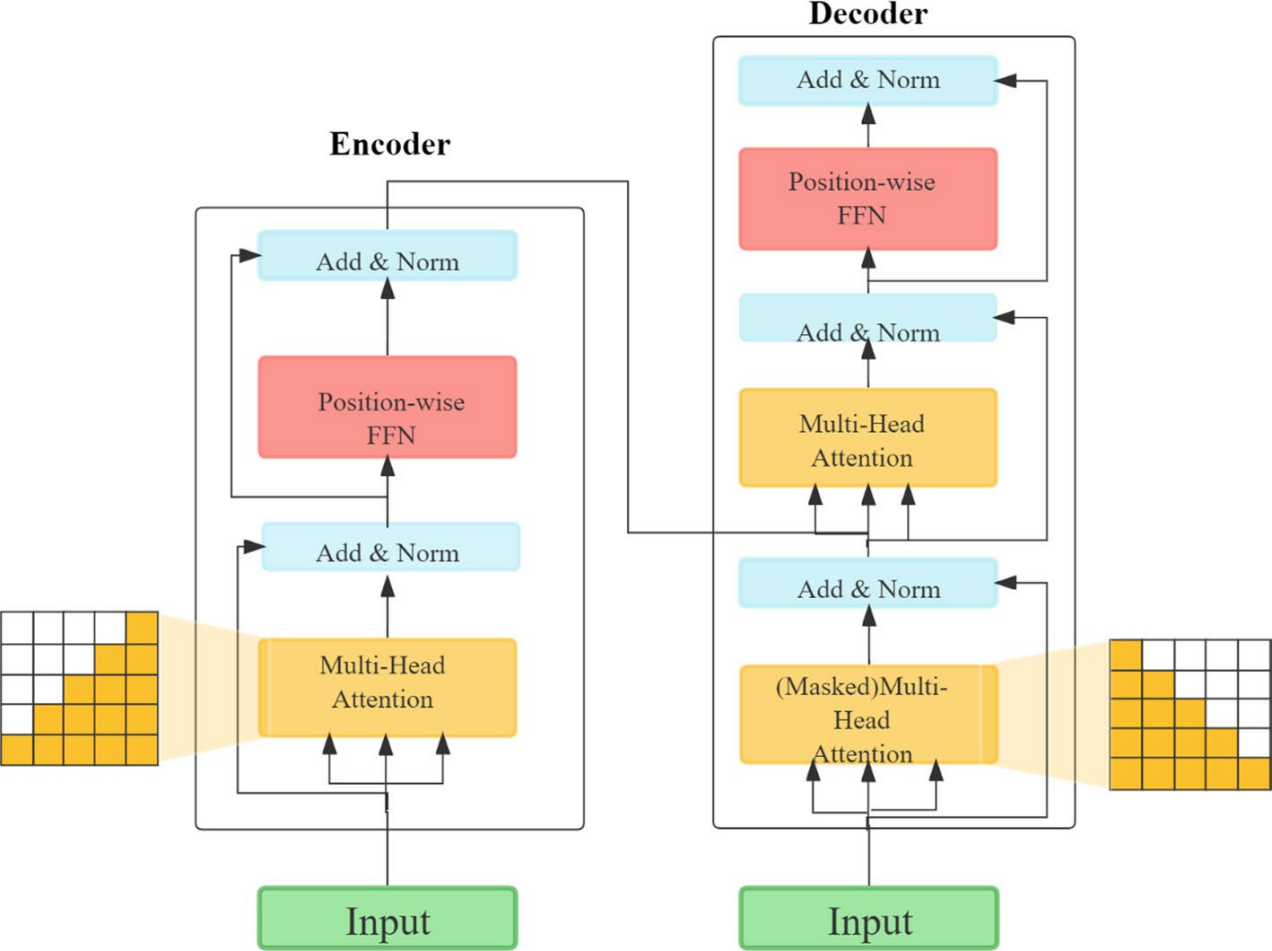
The structure of the encoder and decoder in the transformer model

The canonicalization model used in this work was based on a transformer architecture consisting of two separate stacks of layers for the encoder and decoder, respectively [37]. The structure of the encoder and decoder, as shown in Fig. 2, mainly includes a multi-head attention mechanism layer, a feed-forward fully connected layer and normalization and residual connections [37, 38].

The multi-head attention mechanism incorporates some portion of knowledge written in its internal memory (*V*) with indexed access by keys (*K*). When new data arrive (*Q*), the layer calculates attention and modifies the input accordingly, thus generating the output of the self-attention layer and weighting the parts that carry the essential information. The formulas of *Q*, *K*, and *V* are as follows:

3 $$Q = F_{c} W_{i}^{Q}$$

4 $$K = F_{c} W_{i}^{K}$$

5 $$V = F_{c} W_{i}^{V}$$

$F_{c}$ is the input matrix of the transformer model, $W_{i}^{Q}$ is the query transformation matrix weight vector, $W_{i}^{K}$ is the keyword transformation matrix weight vector, and $W_{i}^{V}$ is the value transformation matrix weight vector.

*Q* and *V* perform dot product operations, and the result is divided by the scaling factor $\sqrt d$ The result is divided by the scaling factor and then multiplied by *V* following the application of the softmax function to obtain the result after self-attention. The output (*H*) of multi-head self-attention is obtained by splicing the self-attention *N-head* times, and the formula for multi-head self-attention is as follows.

6 $$head_{i} = {\text{Multihead}}(F_{c} W_{i}^{Q} ,F_{c} W_{i}^{K} ,F_{c} W_{i}^{V} )$$

7 $${\text{Attention}}(Q,K,V) = {\text{softmax}}\left( {\frac{{QK^{T} }}{\sqrt d }} \right)V$$

8 $$H = {\text{Concat}}(head_{1} ,head_{2} ,...,head_{N - head} )$$

where ${head}_{i}$ denotes the *i-th* self-attention mechanism (*1* <  = *i* <  = *N-head*). LayerNorm [39] indicates layer normalization, which mainly serves to speed up the convergence of the model, while the residual network structure is used to reduce the learning load of the model with the following equation.

9 $$H^{^{\prime}} = {\text{LayerNorm}}(H + {\text{Dropout}}(H))$$

Dropout [40] is a stochastic deactivation strategy to prevent overfitting in models with a large number of parameters. The feed-forward layer enhances the nonlinear capability of the model with two layers of neural networks, and the transformer structure continues after the feed-forward layer with a normalization and residual layer, according to the following equation.

10 $$O = {\text{LayerNorm}}(H^{^{\prime}} + {\text{Dropout}}({\text{Relu}}(H^{^{\prime}} W^{(1)} + b^{(1)} )W^{(2)} + b^{(2)} ))$$

where *O* is the output vector of the transformer layer in the model, *H*′ is the normalized vector, and $W^{\left( 1 \right)}$, $W^{\left( 2 \right)}$, $b^{\left( 1 \right)}$, and $b^{\left( 2 \right)}$ are the weight coefficients and bias of the 2-layer neural network, respectively.

### CNN + Transformer model

**Fig. 3:**
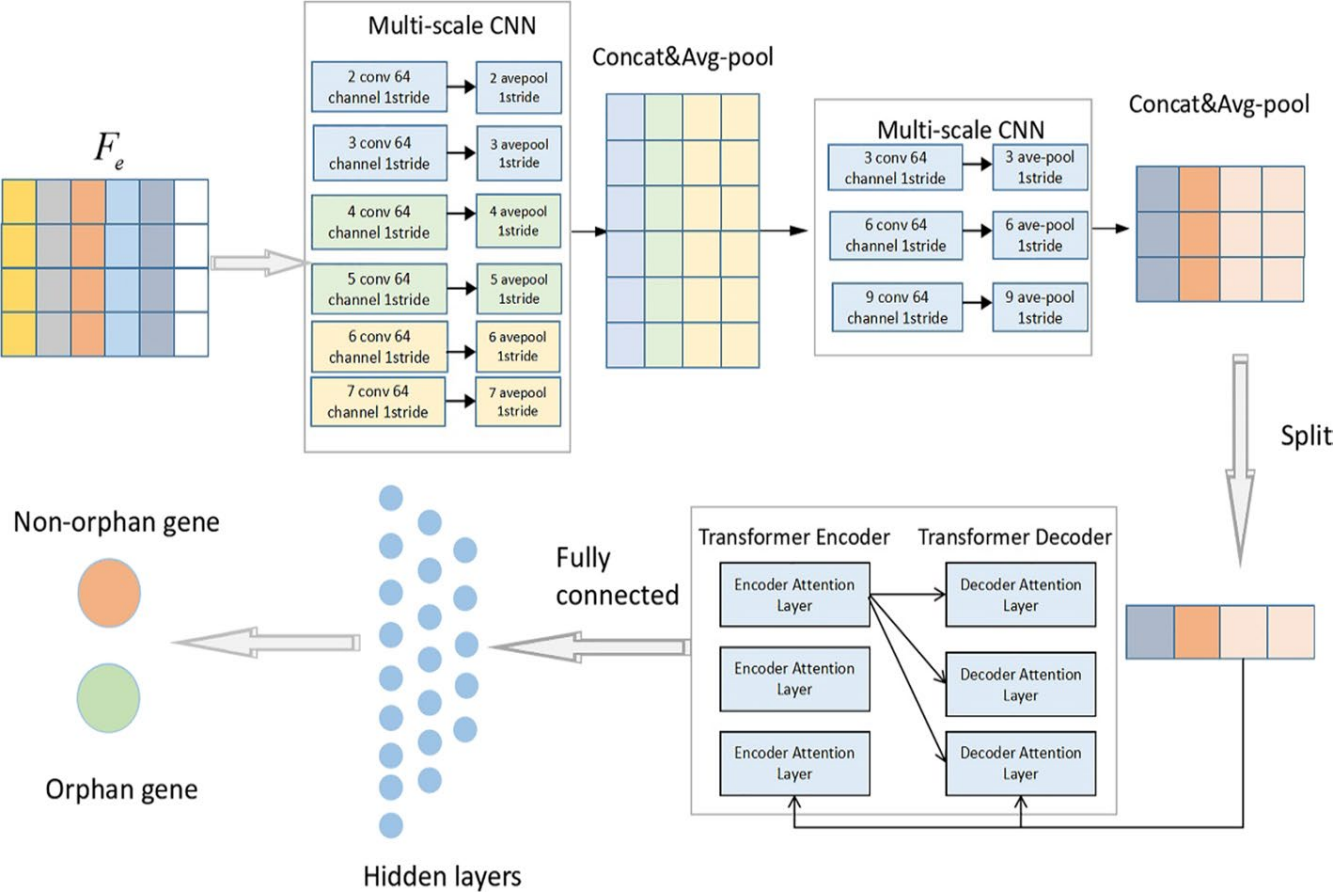
CNN + Transformer model structure. The discrete raw sequence is transformed into a dense, continuous vector F_e_ through feature embedding and then fed into the CNN layer with multi-scale convolution kernels to capture local amino acid k-mers features. The extracted characteristic map of the CNN layer is passed to Transformer neural network. According the multi-head self attention mechanism to capture the long-range interaction characteristics between k-mers. Finally, the Transformer outputs are passed to the fully connected layers to produce identification result

An important disadvantage of the transformer model is its inefficiency in processing long sequences, mainly due to the computation and memory complexity of the self-attention module [41]. CNN can extract local features in the sequence to shorten the length of the sequence [42, 43]. Therefore, this research proposes the CNN + Transformer model structure, which combines a CNN and a transformer model. As shown in Fig. 3, the proposed CNN + Transformer model structure is composed of two multicore convolution layers: a transformer layer and a fully connected layer.

After protein embedding, the protein sequences are encoded as dense, continuous vectors ($F_{e}$) as the input of the CNN layer. The CNN layer in the model consists of two convolutional layers, with the first convolutional layer containing six convolution kernels, as shown in Fig. 3. The variable size of the filter in the convolution is designed to capture k-mer amino acid fragments, where k ranges from 2 (2 peptides) to 7 (7 peptides). The second layer consists of three convolution kernels, denoted as $\left\{ {k_{j}^{n} } \right\}_{n = 1,2,3j = 3,6,9}$, where *n* represents the first few kernels, and j represents the size of the corresponding kernel. The kernel size is equal to the size of a convolutional window across j characters, and the parameters are tuned according to the training and validation step. The intermediate feature map of the *i-th* CNN layer is extracted as $F_{m}^{i} = {\text{Conv}}(F_{e} ,K^{i} )$.

After obtaining the intermediate convolutional feature map ($F_{m}^{i}$), downsampling is performed using AvgPooling by taking the average of the output subregions of the CNN layer, which helps maintain the integrity of the information and facilitates subsequent global feature extraction. After average pooling, the output from all kernels is concatenated for another average pooling operation, which is used to generate the next layer of the feature maps ($F_{c}$), with the following equation:

11 $$F_{c} = {\text{AvgPooling}}({\text{Concat}}({\text{AvgPooling}}(F_{m}^{i} )))$$

where AvgPooling and Concat are average pooling operations and connection operations, respectively.

The transformer layer in the CNN + Transformer model is composed of three transformer encoder-decoder layers. In the transformer layer, the feature representation of long-range interaction information between amino acids is obtained by introducing a multi-head self-attention mechanism, and the values of two hyperparameters in the transformer layer, the number of self-attention heads (N-head) and the number of transformer layers (Num-layer), are discussed and explained in the “Results”.

The output of the transformer layer is flattened to one dimension. The number of output hidden vectors in the fully connected layer is 2, which indicates the binary classification predicted by the model, and the output vector of the binary classification is transformed into the probability *(p)* by the ReLU activation function.

12 $$T^{(1)} = \sigma (W^{l} T^{(l - 1)} + b^{l} )$$

13 $$p = \sigma (W^{s} T^{(l)} + b^{s} )$$

where $\sigma$ is the activation function, $l > = 1$ is the number of layers of the multiconnected neural network, and $W^{l}$ and $b^{l}$ are the connection weights and biases of the hidden nodes from layer $l - 1$ to layer $l$ in the fully connected layer, respectively*.*$W^{l} \in {\mathcal{R}}^{{n_{l - 1} \times n_{l} }}$, $n_{l - 1}$ and $n_{l}$ are the numbers of hidden nodes in layers $l - 1$ and $l$, respectively. $T^{(l)}$ is the output hidden vector of layer $l$. $W^{s}$ and $b^{s}$ are the weights and biases, respectively, of the penultimate layer of the fully connected neural network, and $W^{s} \in {\mathcal{R}}^{{n_{l} \times 2}}$.

### Implementation details

The loss function is cross-entropy loss function and using the Adam optimizer [44]. In the training set, there were approximately 20 times more moso bamboo OG than moso bamboo non-OG, which led to an imbalance problem during training. We explored a cost-sensitive technique for addressing the imbalance problem when training the classifier. The experiments were conducted by adding weights to the different categories of data during training according to the number ratio and using the category weights to train the model. CNN + Transformer involved multiple hyperparameters. These hyperparameters were tuned on the validation set using agrid search procedure. Their optimal values are mentioned below:

1. Embedding dimension: we tested for $e \in \{ 10,20,30,40,50,60,70,80,90,100\}$ and found that the optimal model performance was obtained at e = 50.
2. Convolution filters: at the first convolution layer, we chose six convolution filters, s.t. $f_{k}^{{1}} \in \{ 2,3,4,5,6,7\}$. This allowed us to capture amino acid k-mer frequencies for k-mers of lengths, k = 2 to k = 7. These k-mers represent the local contextual ‘biological’ words. For the second convolution layer, the optimal filter sizes were $f_{k}^{{2}} \in \{ 3,6,{9}\}$. This led to inference of interactions between amino acid k-mers i.e. detect frequencies of local contextual biological phrases consisting of two k-mers having same or different k. For example, the second convolution layer could apprehend interactions between two different dipeptides as well asestimate frequency of a biological phrase comprising a dipeptide and a tripeptide.
3. Transformer encoder-decoder layer number L and self-attention mechanism number H: We took {2, 3, 4} for L and {5, 10} for H, and formed six combinations of L and H, { (2, 5), (2, 10), (3, 5), (3,10), (4,5), (4,10)}. The optimal model performance was attained for L = 3 and H = 10.
4. Fully connected layer dimension: we tested for $f_{c} \in \{ 64,128,256,512\}$ and for optimal model $f_{c}$ was 256.
5. Learning rate: the learning rate for the Adam optimizer was 0.001.
6. Number of epochs: the maximum number of epochs was set to 100 but we enforced early stoppage if the validation loss function stopped improving for two consecutive epochs.
7. Batch size: we tested for batch sizes {64, 128, 256}. The optimal model performance was attained for batch size = 128.

All deep learning models were implemented in Pytorch (1.7.1) [45]. To speed up the training process, a GPU version of PyTorch on an NVIDIA Tesla P100 PCIe 16 GB system was used for the experiments.

### Evaluation strategies

The identification of OG is an imbalance problem, and the number of non-OG in the moso bamboo orphan gene dataset is approximately 20 times greater than the number of OG. Although accuracy and F1 scores are very popular classification evaluation metrics, they can produce misleading results for unbalanced datasets because they do not take into account the ratio between positive and negative samples, and classifiers can achieve good results in terms of specificity but show a large number of false positives. An effective solution for overcoming the class imbalance issue comes from the MCC and BA [46]. When dealing with an unbalanced dataset, GM and BM are better performance metrics if the classification success rate is of concern [47]. Therefore, BA, BM, GM, and MCC were selected as the evaluation metrics in the experiment. The evaluation indicators used in the article are summarized as follows:

14 $${\text{BA = }}\frac{{1}}{{2}} \times \left( {\frac{{{\text{TP}}}}{{\text{TP + FP}}}{ + }\frac{{{\text{TN}}}}{{\text{TN + FN}}}} \right)$$

15 $${\text{GM = }}\sqrt {\frac{{{\text{TP}}}}{{\text{TP + FN}}} \times \frac{{{\text{TN}}}}{{\text{TN + FP}}}}$$

16 $${\text{BM}} = \frac{{{\text{TP}}}}{{\text{TP + FN}}} + \frac{{{\text{TN}}}}{{\text{TN + FP}}} - 1$$

17 $${\text{MCC}} = \frac{{{\text{TP}} \times {\text{TN - FP}} \times {\text{FN}}}}{{\sqrt {{\text{(TP + FP)}} \times {\text{(TP + FN)}} \times {\text{(TN + FP)}} \times {\text{(TN + FN)}}} }}$$

Here, TP is the number of OG identified as OG, FN is the number of OG identified as non-OG, FP is the number of non-OG identified as OG, and TN is the number of non-OG identified as non-OG.

## Results and discussion

### Dataset division

We use 70% of the data for training, 15% of the data for validation, and the remaining 15% of the data as holdout data for testing. We maintain the same ratio between the number of OG and non-OG during the training, validation, and testing of data. Each experiment is executed 10 times to obtain the average performance (i.e., tenfold cross-validation with 15% independent data as testing data for each run). The average performance for the independent holdout testing datasets is reported. The datasets that we use in the experiment are shown in Table 2.

**Table 2:** Division and construction of datasets

Data	OG	Non-OG	Total
Original set	1544	30,443	31,987
Training set	1071	21,317	22,388
Validation set	235	4566	4801
Testing set	238	4560	4798

**Fig. 4:**
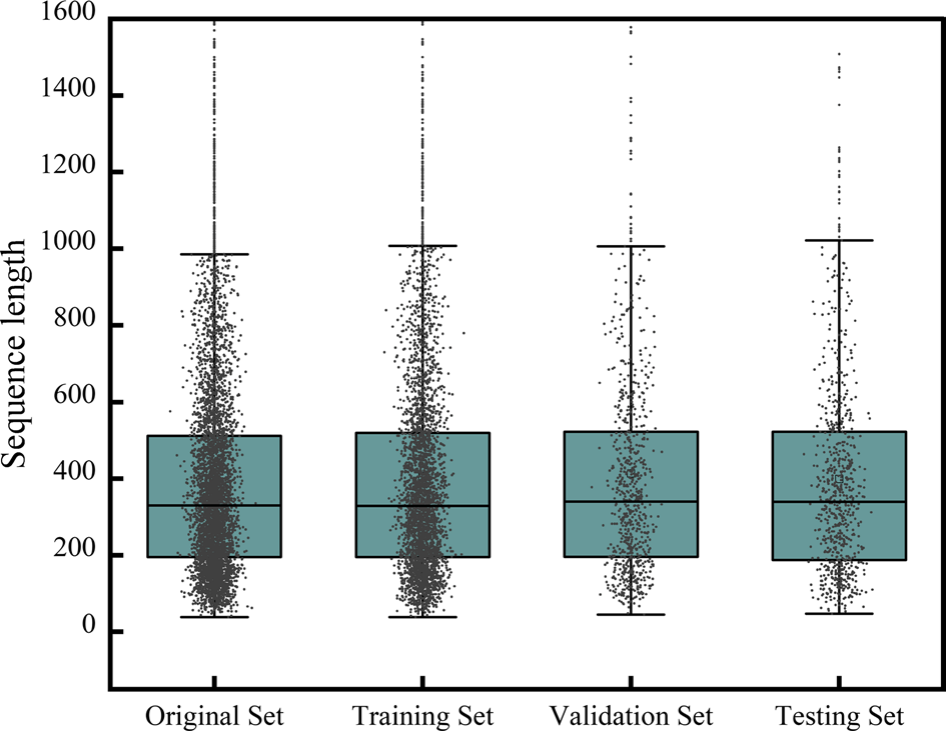
Sequence length distribution in original set, training set, validation set, and testing set

Orphan genes are widely distributed in plant species and generally exhibit significant differences in gene length [2, 10]. As can be seen from Fig. 4, the sequence length distribution of the Original Set, Training Set, Validation Set and Testing Set is similar, indicating that four data sets are comparable.

### Performance comparison of different CNN + Transformer architectures

The CNN + Transformer model structure proposed in this study includes an embedding layer and two multicore convolutional layers: a transformer layer and a fully connected layer. Table 3 shows the performance comparison of CNN + Transformer under different model structures. From the table, we can see that the average BA value and GM value of the proposed CNN + Transformer architecture for the testing set can reach 0.877 and 0.881, respectively. Reducing the CNN layer or transformer layer in this structure will result in a decrease in model recognition performance. Specifically, when the transformer layer is removed from the original structure, the average total BA value and GM value are 0.773 and 0.784, respectively. When two multicore convolutional layers are removed from the original structure, the average total BA value and GM value are 0.844 and 0.832, respectively. In contrast, removing one 3-core convolutional layer or two multicore convolutional layers from the original structure will result in a slight decrease in the recognition performance of the model. After adding a fully connected layer of 256 neurons on the basis of the original framework, the recognition performance of the model is basically the same as that of the original framework, but the complexity of the model structure increases. When a 3-core convolutional layer is added on the basis of the original model structure, the average BA value and GM value are 0.853 and 0.837, respectively, and the recognition performance of the model declines.

**Table 3:** The average BA and GM values under different CNN + Transformer structures

Method	BA	GM	Train time (min)	Test time (s)
E + FC_256	0.677	0.612	25	88
E + CNN_6 + FC_256	0.748	0.644	29	106
E + CNN_6 + CNN_3 + FC_256	0.773	0.784	28	99
E + Transformer + FC_256	0.844	0.832	57	390
E + CNN_6 + Transformer + FC_256	0.866	0.849	61	377
E + CNN_6 + CNN_3 + Transformer + FC_256	**0.877**	**0.881**	44	342
E + CNN_6 + CNN_3 + CNN_3 + Transformer + FC_256	0.853	0.837	48	366
E + CNN_6 + CNN_3 + Transformer + FC_256 + FC_256	0.871	0.865	55	404

Bold values indicate the highest values of different evaluation indicators

E: word embedding coding. CNN_6: multiscale convolution layer, with a convolution kernel size of {2, 3, 4, 5, 6, 7} for each scale. CNN_3: multiscale convolution layer, the convolution kernel size of each scale is {3, 6, 9}. Transformer: three-layer transformer neural network. FC_256: fully connected neural network with 256 neurons

### The effect of hyperparameters on model performance

**Fig. 5:**
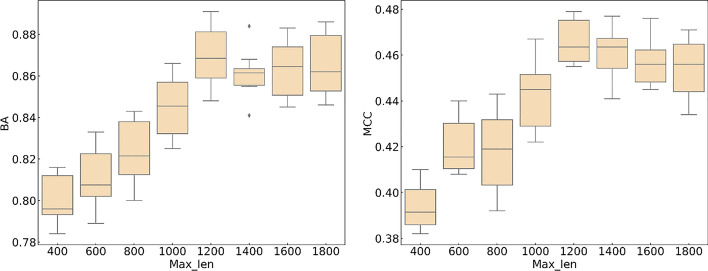
Comparison of CNN + Transformer performance with different Max_len values

We evaluated the robustness of CNN + Transformer and elucidated the effect of two hyperparameters on the model: Max_len and Embedding_dim. The MCC values and balance accuracy were used to evaluate the model as the hyperparameters were adjusted. The first hyperparameter is the maximum sequence length, Max_len. From Fig. 5, it can be seen that when the value of Max_len is less than 1200, the values of MCC and BA are positively correlated with Max_len as a whole. At 1200, the average values of MCC and BA in the model are as high as 0.469 and 0.876, respectively, because some sequences with longer inputs are truncated at shorter lengths, which results in the loss of information contained in the sequences. However, when the sequence length continues to increase, the MCC value and BA value show a slight downward trend, indicating that simply increasing Max_len does not improve the recognition performance of the model but would increase the model recognition time and reduce the model recognition efficiency.

**Fig. 6:**
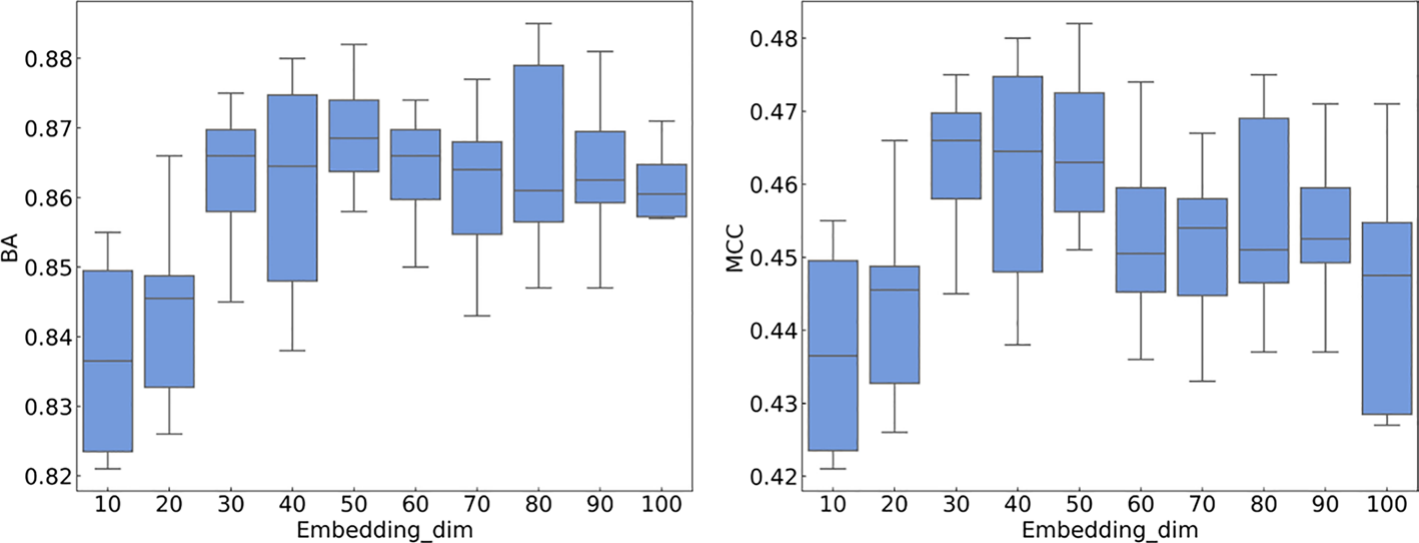
Comparison of CNN + Transformer performance with different Embedding_dim values

The second hyperparameter is the word embedding dimension, Embedding_dim. We take every 10 values from 0 to 100 as the word embedding dimension. As shown in Fig. 6, when Embedding_dim is equal to 50, the best mean MCC values and BA values of the model are highest; the best mean MCC values are 0.481 and 0.467, respectively; and the best mean BA values are 0.892 and 0.876, respectively. When Embedding_dim is equal to 50, increasing Embedding_dim further does not improve the performance of the model but increases the time to train the weights of the embedding matrix. Therefore, an Embedding_dim of 50 is selected for the experiment values.

**Fig. 7:**
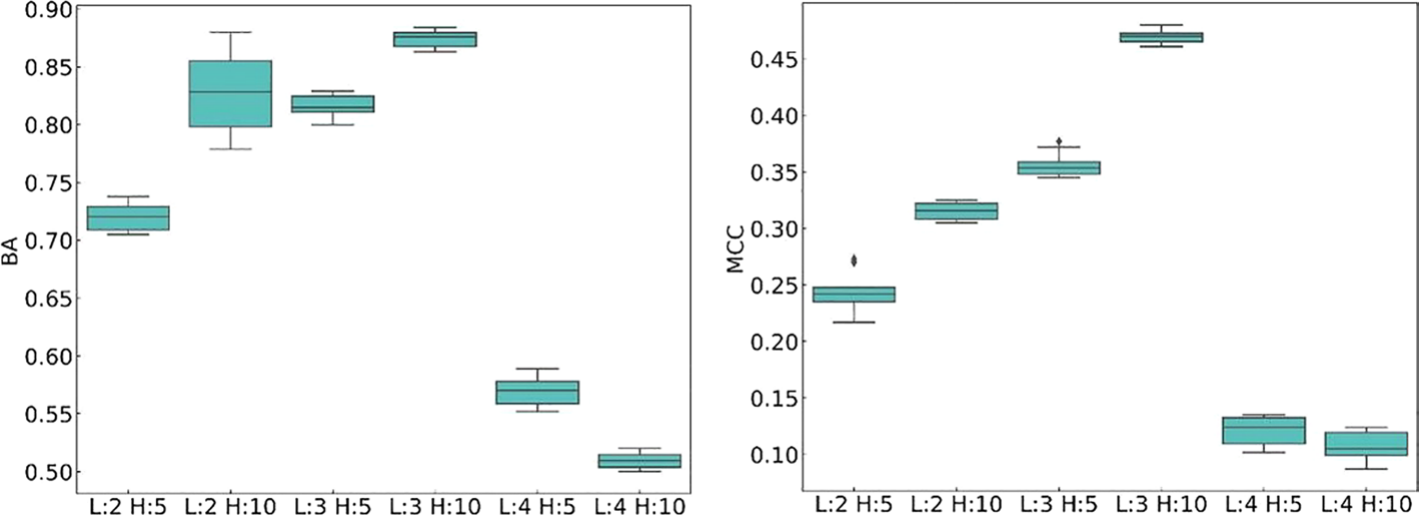
Performance comparison of CNN + Transformer under different N-head and Num-layer combinations. L: transformer encoder-decoder number; H: number of heads of the self-attention mechanism

Next, we study the effect of two other hyperparameters, N-head and Num-layer, in the transformer layer on model performance. Because the head number (N-head) of the hyperparameter multi-head self-attention mechanism must be divisible by Embedding_dim, N-head values of 5 and 10 are chosen for the experiment. The numbers of layers of the encoder-decoder in the transformer hyperparameter are 2, 3, and 4. There are six combinations of N-head and Num-layer. Figure 7 shows the performance comparison of the models in the six different combinations. From the figure, we can see that in the CNN + Transformer model, when there are three layers of the transformer encoder-decoder and 10 heads of the attention mechanism, the best, average, and worst BA and MCC values of the model in the testing set are highest for the six combinations; the best, average, and worst BA values are 0.888, 0.875, and 0.863, respectively; and the highest best, average, and worst MCC values are 0.479, 0.470, and 0.458, respectively. Adding an encoder-decoder layer on this basis will cause the model performance to drop dramatically, with average BA and MCC values of 0.524 and 0.106, respectively. When one layer of the encoder-decoder is reduced or the number of multi-head attention mechanisms is reduced, the recognition performance of the model will also decrease.

### Performance comparison with traditional deep learning models and traditional machine learning models

To verify the recognition performance of CNN + Transformer, we compared it with four basic models (RNN, LSTM, GRU and transformer) that are widely used in deep learning to process sequences and two traditional machine learning models (Support.

Vector Machines (SVM) [48], Random Forest [49]). At the same time, we added the CNN layer in CNN + Transformer to fine-tuned RNN, LSTM and GRU models, and the results are shown in Table 4. Each deep learning model was weighted according to the ratio of OG to non-OG. All models were tested ten times, and the average of the ten test results was used for comparison. As shown in the table, CNN + Transformer performed significantly better than the four basic models according to the four comprehensive indicators of BA, BM, GM and MCC. The MCC value reached 0.471, which was 0.027 higher than the value for of the transformer model, 0.048 higher than that for GRU, 0.053 higher than that for LSTM, and 0.226 higher than that for RNN, and 0.219 higher than that for SVM, and 0.244 higher than that for Random Forest. Compared with Random Forest, SVM, RNN, LSTM, GRU, and the transformer model, the BA values ​​were increased by 0.208, 0.185 0.358, 0.046, 0.037, and 0.031; the GM values were increased by 0.242, 0.212, 0.359, 0.042, 0.037, and 0.033; and the BM values were increased by 0.412, 0.366, 0.712, 0.087, 0.079 and 0.068, respectively. We noticed that the CNN + Transformer model performed better than the transformer model, which proves the importance of the convolution operation in the CNN + Transformer model. Among the basic models, the transformer model showed the best performance, with a balance accuracy of 0.844 and an MCC value of 0.444, which were higher the values for the recurrent neural network. After adding the CNN layer, the recognition performance of the LSTM and GRU models for the moso bamboo orphan gene dataset decreased. Before adding the CNN layer, the average BA values of LSTM and GRU were 0.829 and 0.838, and the average MCC values were 0.418 and 0.423, respectively. After adding the CNN layer, the average BA values of the two models dropped to 0.775 and 0.777, and the average MCC values dropped to 0.376 and 0.373, respectively. However, after the transformer was added to the CNN layer, the model's recognition ability for the moso bamboo orphan gene dataset was improved and the training and testing time of the model was reduced, the balance accuracy and MCC value were increased from 0.844 and 0.444 to 0.875 and 0.471, respectively.

**Table 4:** Model performance comparison

Model	BA	GM	BM	MCC	Train time (min)	Test time (s)
Random forest	0.667	0.629	0.334	0.227	4	22
SVM	0.690	0.659	0.380	0.252	23	77
RNN	0.517	0.512	0.034	0.245	31	123
CNN + RNN	0.503	0.500	0.007	0.109	18	98
LSTM	0.829	0.829	0.659	0.418	36	284
CNN + LSTM	0.775	0.772	0.550	0.376	26	231
GRU	0.838	0.834	0.667	0.423	33	253
CNN + GRU	0.777	0.776	0.554	0.373	24	219
Transformer	0.844	0.838	0.678	0.444	57	387
CNN + Transformer	**0.875**	**0.871**	**0.746**	**0.471**	44	343

Bold values indicate the highest values of different evaluation indicators

In terms of training time and testing time, the Transformer model has slightly higher training and testing time than the recurrent neural network model, which is caused by the computational complexity of multi-head self-attention mechanism in Transformer. Although the complexity of CNN + Transformer model structure is higher than that of Transformer model, the training and testing time of CNN + Transformer model is lower than that of Transformer model. Because the one-dimensional convolution layer of CNN + Transforme model performs preliminary feature extraction on the input feature matrix, the size of the feature matrix is compressed, thus reducing the computational complexity of the model. These results further prove that CNN + Transformer is an effective deep learning model for moso bamboo OG recognition.

### CNN + Transformer model verification

The genome of moso bamboo has been updated to the second edition, which includes 50,936 protein sequences [50]. The model is tested on the dataset of second edition. Firstly, we obtained 1275 orphan genes (Additional file 2: Table S2) from the second edition of moso bamboo protein sequences through BLAST sequence alignment. Then, we input all the protein sequences of moso bamboo into the CNN + Transformer model, and the model identified 1466 orphan genes of moso bamboo.

In order to verify the reliability of CNN + Transformer model in identifying orphan genes of moso bamboo, we compared the 1466 orphan genes with 1275 orphan genes which were obtained by BLAST method. The results showed that 1106 protein sequences (Additional file 3: Table S3) were identical and our method had a high coincidence with BLAST results. To further validate the performance of CNN + Transformer model, we trained an optimal CNN + Transformer model using 70% data for training, 15% data for verification and 15% data for testing in the second version of moso bamboo orphan genes dataset. The test set contained 194 orphan genes identified by BLAST tools. The CNN + Transformer model identified 211 protein sequences as orphan genes in the test set, 183 of which were coincident with BLAST results. The above results indicated the reliability of CNN + Transformer in identifying orphan genes of moso bamboo.

### OGs functional analyses

**Fig. 8:**
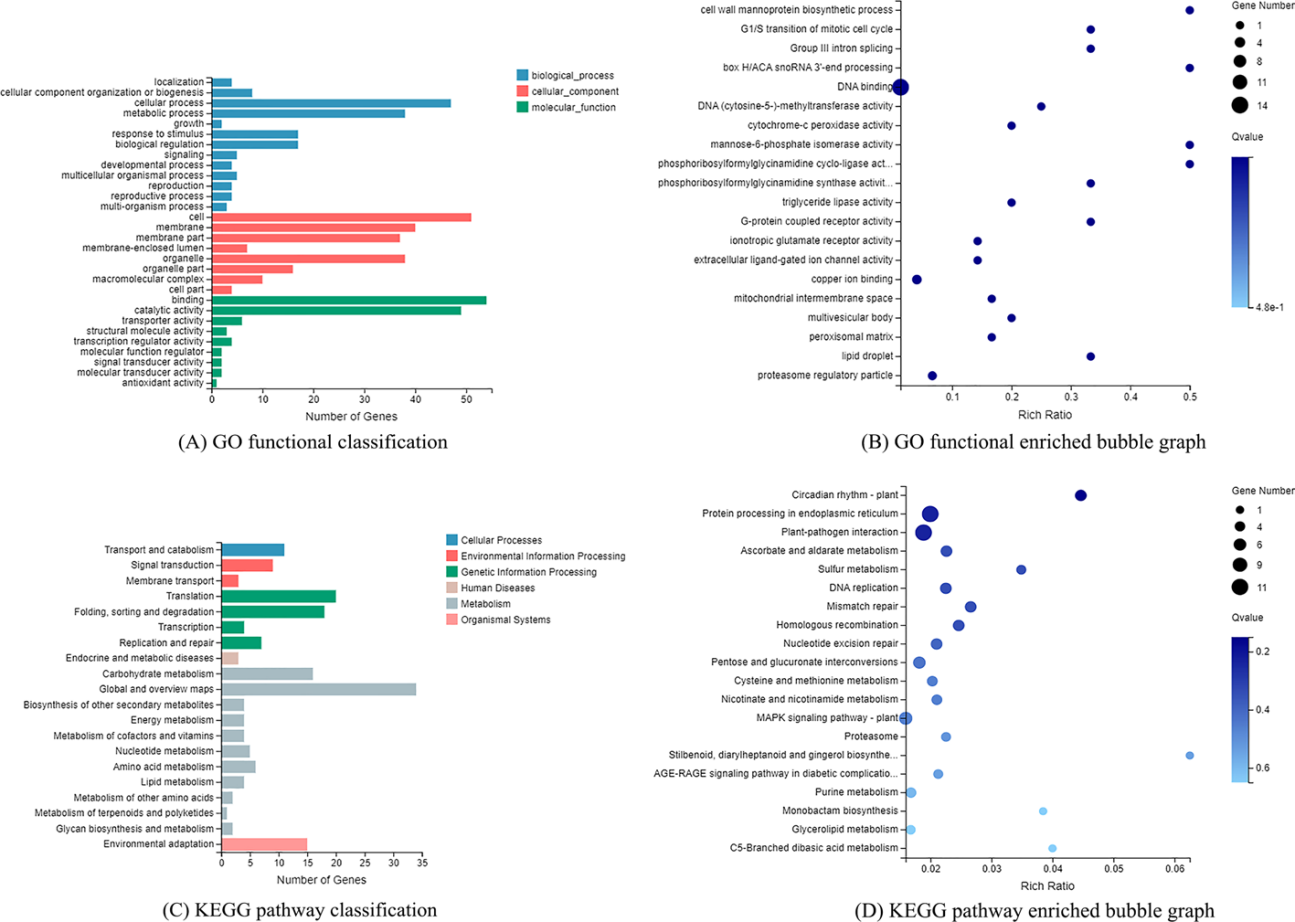
Functional classification and enrichment of OGs (Dr.Tom, BGI, China). **A** GO (Gene Ontology) classification of OGs. The vertical axis represents the GO terms, and the horizontal axis represents the number of OGs. **B** Bubble graph for GO enrichment (the bigger bubble means the more genes enriched, and the increasing depth of blue means the differences were more obvious; q-value: the adjusted *p*-value). **C** KEGG (Kyoto Encyclopedia of Genes and Genomes) classification of OGs. **D** Bubble graph for KEGG enrichment

Functional annotation, classification and enrichment (GO, KEGG) analysis were performed by the BGI in-house customized data mining system called Dr.Tom (http://report.bgi.com). The 1254 OGs of moso bamboo were searched against the GO database in order to categorize standardized gene functions. Some OGs were classified into “cellular process”, “metabolic process”, “catalytic activity”, “binding”, and “cell” (Fig. 8 (A)). In Fig. 8 (B), we performed GO enrichment analysis on OGs, functions such as “cell wall mannoprotein biosynthetic process”, “box H/ACA snoRNA 3’-end processing”, “mannose-6-phosphate isomerase activity” and “phosphoribosylformylglycinamidine cyclo-lingase activity” were enriched. According to KEGG pathway annotation, the KEGG pathway classification graph (Fig. 8 (C)) and enrichment graph (Fig. 8 (D)) are generated [51–53], phyper function in the R project was used to calculate *P* values and false discovery rates (FDRs). The 1254 OGs were divided into several categories (Fig. 8 (C)). Among them, “Translation”, “Folding, sorting and degradation”, “Carbohydrate metabolism” and “Environmental adaptation” were the most prominent. In Fig. 8 (D), we performed KEGG enrichment analysis on OGs, pathways such as “Circadian rhythm-plant”, “Protein processing in endoplasmic reticulum”, and “Plant-pathogen interaction” were enriched.

## Conclusion

Using the sequence alignment method to identify OG in species is time-consuming and laborious, so it is a great challenge to design a robust and efficient model for identifying OG in species. In this study, we propose the sequence-based deep learning model CNN + Transformer with the aim of exploring whether deep learning shows better performance in the identification of moso bamboo OG (an unbalanced classification problem). The model uses a CNN to capture local k-mer amino acid features in the protein sequence and a transformer model to capture remote features between k-mer amino acids. CNNs are often used to capture local features, but they show some defects in effectively identifying the interdependence among long-distance input data. In contrast, in the model based on the transformer neural network, the long-term dependency relationships between local features are captured by introducing a multi-head self-attention mechanism.

The performance of CNN + Transformer was evaluated with a moso bamboo orphan gene dataset, and it achieved very good performance according to four comprehensive evaluation indexes: BA, GM, BM and MCC. Compared with four other models (RNN, LSTM, GRU, and transformer) that are widely used to address sequence problems in deep learning, the performance of CNN + Transformer was significantly superior, which further proved that CNN + Transformer is an effective gene recognition model for moso bamboo OG. At the same time, we combined the CNN layer of CNN + Transformer with RNN, LSTM, GRU and other models and made fine adjustments. The results showed that the recognition performance of the RNN, LSTM and GRU models declined to varying degrees after adding the CNN layer. The efficiency of the transformer model in capturing the correlation dependence between k-mer amino acids in the protein sequence was verified. Subsequently, we compared the results of CNN + Transformer and BLAST on moso bamboo orphan gene dataset of the second edition, and verified that CNN + Transformer is a reliable orphan gene identification model of moso bamboo.

CNN + Transformer model was used to predict orphan genes directly from protein sequences, which was essentially different from BLAST method. Therefore, when researchers want to know whether some genes are orphan genes, CNN + Transformer can assist researchers to further confirm orphan genes as an effective tool. In the future, we will explore and integrate orphan gene data of other species to further improve the performance of CNN + Transformer. At the same time, we are interested in how to use deep learning to automatically learn features from biological data rather than manually extracting features heavily based on domain knowledge.

## Supplementary Information

**Additional file 1.** Includes 1544 orphan genes of the first version moso bamboo.

**Additional file 2.** Includes 1275 orphan genes of the second version moso bamboo

**Additional file 3.** Includes 1106 the second edition orphan genes of moso bamboo further screened by CNN+Transformer model.

## Data Availability

The datasets generated during and analysed during the current study and the source code can be downloaded from Github (https://github.com/xuan2333/CNN+Transformer).

## Abbreviations

CNN: Convolutional neural network
ECs: Evolutionarily conserved genes
GO: Gene Ontology
GRU: Gated recurrent unit
KEGG: Kyoto Encyclopedia of Genes and Genomes
LSTM: Long short-term memory
OG: Orphan genes
RNN: Recurrent neural network
SVM: Support Vector Machines
